# Molecular Mechanisms of Cancer Drug Resistance: Emerging Biomarkers and Promising Targets to Overcome Tumor Progression

**DOI:** 10.3390/cancers14071614

**Published:** 2022-03-23

**Authors:** Fabrizio Fontana, Martina Anselmi, Patrizia Limonta

**Affiliations:** Department of Pharmacological and Biomolecular Sciences, University of Milano, 20133 Milano, Italy; fabrizio.fontana@unimi.it (F.F.); martina.anselmi@unimi.it (M.A.)

Cancer still represents a major global burden, being the second leading cause of death worldwide [1]. Standard cancer treatments include surgery, radiotherapy and chemotherapy [2,3]. Targeted therapies (small molecules and monoclonal antibodies) have also been developed; these treatment approaches are based on drugs that target specific signaling pathways involved in cancer development and progression [4,5,6]. More recently, the field of cancer treatment has been revolutionized by the development of immunotherapy (i.e., immune checkpoint inhibitors) based on the aim to boost patients’ own immune systems to fight cancer, although several immune-related side effects were reported to be associated with this therapeutic approach [7,8].

Unfortunately, treatment failure followed by cancer recurrence is a common hallmark of most tumors, due to the development of drug resistance. This Special Issue was foreseen to highlight and discuss the most recent advances in the molecular mechanisms involved in the progression of cancers towards their most aggressive resistant stage.

Taxanes (paclitaxel, docetaxel, cabazitaxel) are a well-known family of chemotherapeutic drugs still widely used to treat different types of cancers (mainly epithelial-derived cancers), based on their ability to suppress microtubule dynamics, thus blocking mitosis and triggering apoptosis in tumor cells [9]. Unfortunately, despite their initial strong efficacy in reducing tumor growth, most patients experience the development of resistance to these drugs. In their review, Maloney et al. [10] provide a comprehensive overview of the molecular mechanisms underlying the development of resistance to taxane in different tumors. These mechanisms include: upregulation of pro-survival, anti-apoptotic and epithelial-to-mesenchymal transition (EMT)-inducing pathways; increased activity of drug export transporters, responsible for decreased intracellular drug levels; and increased taxane metabolism. The involvement of non-coding RNAs in these resistance-related mechanisms is also discussed in this article.

Based on the peculiar role of receptor tyrosine kinases (RTKs) in tumor development and progression towards its most aggressive phase, tyrosine kinase inhibitors (TKIs) were developed and introduced, in the form of an oral therapy, in the clinical settings for the treatment of different types of cancer, being referred to as “targeted therapies”. It is well accepted that anticancer compounds must be efficiently absorbed at the gut level to enter the circulation and, finally, to reach the tumor cells. Taking advantage of the optimization of the CaCo2 gut epithelial model, Honeywell and coworkers demonstrate that different TKIs are poorly absorbed by these cells, due to the drug transport systems expressed at their membrane level and the intracellular presence of specific metabolizing enzymes. These data support that a low bioavailability of these compounds might deeply contribute to the acquisition of the TKI-resistant phenotype observed in different types of tumors [11].

Oncogenes were also reported to mediate the development of chemotherapy resistance in cancer cells. In this issue, Manna and Sarkar summarize and discuss the multifaceted role of the oncogene Astrocyte elevated gene 1/Metadherin (AEG-1/MTDH) in the promotion of chemoresistance. Specifically, AEG-1 favors the translation of the ATP binding cassette transporter MDR1/ABCB1 mRNA, boosting doxorubicin efflux from cancer cells. AEG-1 stimulates the synthesis of dihydropyrimidine dehydrogenase, the enzyme responsible for the metabolism of the chemotherapeutic drug 5-FU (5-fluorouracil) and induces the transcription factor late SV40 factor (LSF), resulting in an increase in thymidylate synthase, the substrate of 5-FU. This oncogene triggers the activation of the AMPK/mTOR signaling pathway, leading to protective autophagy and resistance to both doxorubicin and 5-FU, and interferes with the binding of retinoic acid to its specific receptor (RXR), thus inhibiting the expression of its specific target genes, and subsequently promoting retinoic acid resistance. AEG-1 also triggers chemotherapy resistance by inducing the expression of the MET proto-oncogene, as well as the ALDH3A1 enzyme (aldehyde dehydrogenase 3 family member A1) [12].

Another oncogene shown to be deeply involved in the development of drug resistance in common solid and haematological tumors is the tyrosine kinase receptor Axl. This gene was found to be overexpressed in drug-resistant cancer tissues and to mediate tumor adaptation to standard therapies through the activation of the intracellular signaling pathways associated with RTKs, such as EGFR (epidermal growth factor receptor), HER2 (human epidermal growth factor receptor 2) and HER3, as discussed by Wium and coworkers [13].

The presence of a functional vasculature is a well-established hallmark of cancers, classically thought to be required to sustain tumor growth and dissemination. More recently, a more complex and multifaceted role of tumor vascularization has emerged, based on the observation that antiangiogenic drugs, instead of inhibiting cancer growth, foster cancer cells towards increased blood vessel formation, leading to recurrence and development of drug resistance. In their article, Belotti et al. address the issue of the different mechanisms underlying vascularization in tumors (vasculogenesis, glomeruloid proliferation, intussusceptive angiogenesis, vasculogenic mimicry and vessel co-option), and how these mechanisms may influence the tumor response to anticancer treatments; the role of alternative mechanisms of therapy-induced vessel formation in tumor recurrence and development of drug resistance is also comprehensively discussed by these authors [14].

Cancer stem cells (CSCs) are a very aggressive subpopulation of cells in the tumor mass, characterized by self-renewal, tumor-initiating capacity and adaptive abilities. Accumulating evidence strongly supports that CSCs play a key role as a drug resistance and tumor relapse driving factor. The presence of these cells was reported to correlate with poor overall- and disease-free survival, as well as with tumor progression and recurrence. Marzagalli and coworkers provide an in-depth discussion about the different molecular mechanisms (growth factors, their receptors and intracellular signaling pathways) which mediate the ability of CSCs to escape the antitumor activity of chemotherapeutics, targeted therapies and immunotherapies, highlighting their plasticity as an insidious feature responsible for the development of drug resistance in different types of tumors [15].

Extracellular vesicles (EVs) are nano-sized vesicles deeply involved in the intercellular communication in different tissues. In tumor tissues, they were widely reported to carry different molecular cargos (proteins, mRNAs, microRNAs) from cancer cells to cells in their microenvironment, and vice versa, thus mediating a vicious cell-to-cell cross-talk. In their review article, Fontana and coworkers summarize the most relevant recent findings about the role of EVs in the acquisition of the drug-resistant phenotype in cancer. First, EVs were shown to trigger chemoresistance through direct loading and expulsion of the drugs, also favored by the presence of ATP-binding cassette pumps in their membranes. Interestingly, EVs are also utilized by cancer cells to transmit drug resistance to their neighboring drug-sensitive cancer cells, via a horizontal transfer of drug efflux pumps, as well as of pro-survival proteins and RNAs. More recently, cancer-derived EVs have been widely shown to transfer specific molecular cargos to stromal cells (i.e., adipocytes, fibroblasts, immune cells) in their microenvironment, shaping these cells towards a protumoral phenotype. Being easily detectable in liquid biopsies, particularly in plasma, EVs have a great potential as novel and valuable diagnostic/prognostic markers in different types of cancer [16].

The peculiar, recently reported, cell-to-cell communication between cancer cells and the neurons in their microenvironment is discussed by Hunt et al. Tumor cells secrete growth factors (i.e., nerve growth factor, NGF) and microRNAs (miRNAs) that educate neurons towards a protumoral phenotype. Moreover, cancer cells express chemokine receptors, such as the CCR2 receptor, which is activated by its neuron-derived specific ligand CCL2; activation of this CCR2/CCL2 axis is in turn responsible for the outgrowth of neurites towards the cancer site. Neurotransmitters and neuropeptides are also released from neurons to favor tumor progression, while cancer cells secrete axon guidance molecules, directing nerve outgrowth in the tumor microenvironment. Interestingly, EVs and their molecular cargo (specifically miRNAs) are also deeply involved in this neurons-tumor cells communication. Based on these observations, the authors conclude that a better understanding of this cross-talk might represent a target for promising anticancer therapies aimed at overcoming treatment resistance in solid tumors [17].

Different articles in this Special Issue specifically address the molecular mechanisms underlying the development of drug resistance in hormone-related tumors, such as breast, ovarian and prostate cancer.

In breast cancer, Tsoi and coworkers report that BQ323636.1, a splice variant and inhibitor of the co-repressor protein NCOR2, favors the binding of the estrogen receptor (ER) to the IL-6 promoter and upregulates both IL-6 and IL-6 receptor (IL-6R) expression, leading to the activation of STAT3. Targeting the IL-6/STAT3 signaling pathway, through IL-6R silencing or treatment with the specific IL-6R antibody tocilizumab, effectively reverses tamoxifen resistance in breast cancer cells, both in vitro and in vivo [18]. An altered signaling from the HER receptor family is also deeply involved in the development of drug resistance in breast cancer. Lapatinib, a specific HER2 inhibitor, is widely used as a treatment for HER2+ breast cancer patients; however, the prognosis is poor since most patients acquire resistance. Lee et al. demonstrate that the Heat Shock Protein 90 (HSP90) is significantly increased in drug resistant breast cancer cells. These authors propose that a combination of lapatinib and HSP90 inhibitors may represent a novel promising therapeutic strategy for HER2+ patients [19]. An additional mechanism involved in the acquirement of resistance against anti-HER2 therapies is the increased expression of the HER3 receptor isoform. In their article, Cruz and coworkers provide evidence that, in breast cancer cell and tissue models, the junctional adhesion molecule-A (JAM-A) upregulates the expression of HER3 via a molecular pathway involving β-catenin and FOXA1. These data support that JAM-A deserves to be considered an effective target to prevent the development of resistance to HER2-targeted therapies [20]. The presence of aberrant alterations of the cellular metabolism is now widely accepted as a peculiar hallmark of tumors, including breast cancer. Specifically, increased glucose uptake, hyperactivated glycolysis and dysfunctional oxidative phosphorylation have been shown to be deeply associated with cancer progression towards the most aggressive phases and the development of drug resistance to standard drugs, such as cisplatin, paclitaxel, tamoxifen and doxorubicin. In this Special Issue, Varghese et al. provide a comprehensive review of the current knowledge on novel therapeutic strategies, specifically targeting the aberrant glucose metabolism in drug-resistant breast cancers (i.e., 2-deoxy-D-glucose, metformin, phytochemicals) [21]. The presence of a vicious cross-talk between cancer cells and cells in their microenvironment, such as fibroblasts, adipocytes and immune cells, is now well established and demonstrated to play a key role in the development of resistance to anticancer therapies. Different soluble biofactors, as well as extracellular vesicles, were demonstrated to be deeply involved in this deleterious communication in breast cancer. Specifically, the review by Cosentino and coworkers addresses in detail the peculiar role of miRNAs in mediating both the induction of protumoral features in stromal cells and the stromal cell-mediated fostering of cancer aggressiveness and progression towards its drug-resistant phase [22].

Ovarian cancer still represents one of the most common causes of death among gynecological tumors, due to its aggressive features and rapid development towards the drug-resistant stage. In their review, Seborova et al. address the molecular mechanisms (i.e., regulatory elements from the non-coding RNA families) involved in these processes. Specifically, these authors summarize and discuss the current available findings highlighting the peculiar role of miRNAs and long non-coding RNAs in the metastatic spread of ovarian cancer, thus representing a promising tool for monitoring the patient’s response to therapies [23]. Platinum-based chemotherapy, such as cisplatin, represents the standard-of-care treatment for ovarian cancer; however, toxicity and acquired resistance to this drug have proven challenging in the treatment of patients with ovarian cancer. Thus, the identification of molecular biomarkers to predict the response to cisplatin would be beneficial to develop novel effective therapies. The involvement of a tumor’s intrinsic DNA repair capacity in drug resistance is now widely accepted. In their original article, Guffanti and coworkers investigate whether the expression of nucleotide excision repair (NER) proteins (ERCC1, XPF, ERCC1/XPF complex) and of the base excision repair (BER) protein DNA polymerase β could represent a possible biomarker of cisplatin response in a platform of established patient-derived ovarian carcinoma xenografts. However, they report that none of these proteins could predict cisplatin activity in the ovarian cancer models. The authors conclude that DNA functional assays might represent a more reliable method to predict the response to platinum-based therapy in ovarian cancer [24].

Prostate cancer is androgen-dependent in its early stages, and androgen receptor (AR) inhibition (androgen deprivation therapy, ADT) still represents the standard treatment for PCa patients. Unfortunately, the tumor often progresses towards its most aggressive, castration-resistant (CRPC), stage. The review article by Ehsani et al. dissects the acquired and intrinsic molecular mechanisms that are involved in the development of PCa towards CRPC and contribute to drug resistance. These mechanisms include: AR gene amplification, mutations and AR splice variants; increased activity of AR co-regulators; non-genomic activities of AR; mutations of the *PTEN* tumor suppressor gene; alternative and intratumoral androgen biosynthesis; autophagy; and expression/activity of the glucocorticoid receptor, as well as of oncogenes/tumor suppressor genes (*N-Myc*, *ONECUT2*, *p53*, the AKT-mTOR pathway, miRNAs and long non-coding RNAs) [25]. An overview of the miRNAs involved in PCa development is provided by Doldi and coworkers; specifically, these authors provide a comprehensive list of the main PCa-related miRNAs and the specific molecular mechanisms by which they contribute to PCa response to radiation and drug therapy [26].

This Special Issue also discusses in detail the molecular mechanisms of drug resistance in different tumors that are not related to the reproductive tissues, since failure of therapeutic approaches represents an unfavorable distinct feature of most, if not all, types of cancers.

The breast cancer resistance protein (BCRP or ABCG2) is a xenobiotic transporter involved in the mechanisms of multidrug resistance, being responsible for the efflux of many anti-cancer drugs. By using melanoma-bearing mice as the experimental model of their study, Szczygiel and coworkers demonstrate that the subcutaneously growing tumor is associated with the upregulation of BCRP expression, evaluated by immunohistochemitry and qRT-PCR, in different hosts’ normal tissues and organs. Since this mobilization of the transporter occurs already in the early stages of tumor development, the authors suggest that this mechanism might be responsible for the induction of primary multidrug resistance [27]. An interesting additional mechanism that might play a key role in melanoma progression is proposed by Appleton et al. in their original article. Most melanomas (about 50%) harbor BRAF^V600^ mutations, while 30% are driven by NRAS mutations. Targeted drugs (i.e., vemurafenib, dabrafenib, trametinib, cometinib) represent the therapy of choice for BRAF mutant melanoma patients; however, these drugs are not effective in NRAS mutant tumors. The authors report that treatment of NRAS mutant melanoma cells with inhibitors of mutated BRAF leads to an increased activation of the Rho/MRTF pathway. A combination treatment of trametinib with CCG-22274 (a Rho/MRTF pathway inhibitor) synergistically reduces cell viability, induces apoptosis and reduces clonogenity in melanoma cells resistant to trametinib. These data support a deep involvement of the Rho/MRTF pathway in the intrinsic resistance to BRAF mutant-targeted drugs in melanomas harboring the NRAS mutation [28].

EGF receptor (EGFR) activating mutations frequently occur in non-small cell lung cancer (NSCLC). Osimertinib, an irreversible EGFR tyrosine kinase inhibitor, has shown significant clinical benefits for NSCLC patients with EGFR activating mutations; however, resistance to this drug is a very common event in these patients. La Monica and coworkers demonstrate that the CDK (cyclin-dependent kinase) 4 and 6 inhibitor abemaciclib markedly inhibits cell growth, spheroid formation, and colony formation in a panel of NSCLC cell lines made resistant to osimertinib. Moreover, in osimertinib-sensitive NSCLC cells, a combination of abemaciclib with osimertinib significantly prevents the onset of resistance. The authors propose that a combination of the two drugs might represent a novel approach to prevent osimertinib resistance in NSCLC patients [29].

The key role of the vicious cross-talk between cancer cells and cells in the tumor microenvironment (TME) in cancer development and progression has been addressed above. In their review article, Domen and coworkers discuss the different mechanisms of the role of cancer-associated fibroblasts (CAFs) in tumor progression and development of drug resistance in lung and pancreatic cancer. Specifically, in both tumors, CAFs have been widely reported to trigger tumorigenic effects through the release of different cytokines, chemokines and growth factors, as well as through the activation of pro-survival pathways in target cancer cells. Based on their ability to affect the response to clinical therapies, CAFs might represent an effective molecular target for novel treatments in lung and pancreatic cancer. The CAF-directed anticancer therapies already investigated in clinical trials and those currently under clinical investigation are discussed in this review [30].

The current standard treatment of locally advanced rectal cancer is based on neoadjuvant chemotherapy followed by surgery; however, only 30% of patients face a complete response. It is now established that both activation and inactivation of cancer-associated genes can occur by epigenetic mechanisms. In particular, DNA methylation, among the other epigenetic mechanisms (histone deacetylation, chromatin remodeling, small noncoding RNA expression), has been demonstrated to be a major player in the molecular mechanisms that influence tumor progression and response to therapy. In their original article, do Canto and coworkers investigate the DNA methylation markers that might represent effective biomarkers of predictive complete response (pCR) in locally advanced rectal cancer (LARC) patients treated with neoadjuvant chemotherapy. Specifically, by using a throughput DNA methylation analysis, they use pre-treatment biopsies to evaluate the predictive relevance of differentially methylated CpGs changes. They report that three CpG-rich islands linked to *OBSL1*, *GPR1* and *INSIG1* genes are able to specifically discriminate pCR from incomplete responders. By exploring the genomic context of these genes, the authors provide evidence that they play a role in gene expression regulation and conclude that the three identified CpGs, which can be easily evaluated in the clinical practice, might represent a novel predictive tool for a pre-treatment screening, useful for the identification of the appropriate treatments for LARC patients [31].

High-grade osteosarcoma (HGOS) still represents the most common primary malignant tumor of the bone; the standard treatment strategies for this tumor are associated with a low cure rate (approximately 40–50%), due to the development of intrinsic or acquired drug resistance. In this issue, Hattinger et al. address the drug-resistance-related biomarkers recently indicated to be involved in HGOS. Specifically, consolidated biomarkers of resistant HGOS include: drug efflux transporters, DNA repair factors, methotrexate resistance-related factors, EVs, non-coding RNAs, and CSCs. The authors discuss the role of recently developed non-conventional treatment strategies (i.e., novel inhibitors of drug efflux transporters, synthetically modified conventional drugs such as doxorubicin, nanocarriers and nanoparticles designed to specifically deliver conventional chemotherapeutic drugs to HGOS cells) targeting these biomarkers with the aim to overcome drug resistance in this aggressive tumor [32].

The key role of the human gut microbiota in the development and progression of different oncological diseases is now well accepted [33]. Specifically, alterations of the gut microbial community (dysbiosis) have been demonstrated to be deeply involved in colorectal cancer (CRC) development and progression to its chemoresistant stage [34]. The use of gut bacteria-related biomarkers to predict the response to therapy in this tumor has been gaining interest and relevance in the last years. The role of the dysbiotic microbiota in the response of CRC patients to anticancer therapies is discussed by Veziant et al. These authors address the role of different bacterial species, such as *F. nucleatum*, *B. fragilis* and colibactin-associated *E. coli* (CoPEC) as effective biomarkers for CRC screening and for the prediction of prognosis and/or treatment response. In particular, CoPEC bacteria is the prevailing species in the colonic mucosa of CRP patients and trigger colorectal tumorigenesis by causing DNA double-strand breaks, increased ROS production, as well as chromosomal instability. These observations support the notion that CoPEC might be considered both a factor predictive of poor outcomes and a possible effective therapeutic target in CRC [35].

Glioblastoma (GBM) is a deadly malignancy, mainly due to its frequent and rapid acquirement of a drug-resistant phenotype; a high failure rate of response has also been reported for recently developed anticancer drugs. Lyne and Yamini carry out a systematic analysis of the data from the literature, as well as from clinical trials sustaining the use of repurposed drugs for the treatment of GBM patients; these drugs include: antineoplastics, disulfiram, antimalarials, anti-inflammatories, immunosuppressants, checkpoint inhibitors, diabetic agents, and small molecules. According to the collected data, improved effects can also be observed when the repurposed agents are combined with each other or are added to the current standard-of care regimens. The authors conclude that repurposing can represent a cost-effective approach to identify drugs to be used in multimodal strategies, with the aim to increase drug response while escaping drug resistance in GBM patients [36]. The TME is deeply involved in the mechanisms of drug resistance also in GBM, as discussed above for other cancers. Specifically, resistance to immunotherapy is one of the peculiar mechanisms shown to be involved in the acquisition of treatment resistance in GBM tumors. Miyazaki and coworkers highlight that the main cause of GBM recurrence after a therapy based on immune-checkpoint inhibitors may be the presence of an immunosuppressive TME, that involves cytokines, chemokines, and EVs produced by tumors as well as by immunosuppressive cells. Among these cells, M2 macrophages and myeloid-derived suppressor cells have been reported to be the master players in the deleterious TME-tumor cross-talk in GBM. In this review article, the authors dissect in detail the tumor immune microenvironment, as well as the correlation between the expression of immune-checkpoint molecules and the prognosis of GBM. The different therapeutic strategies that might overcome these mechanisms by targeting immunosuppressive cells (M2 macrophages, myeloid-derived suppressor cells, regulatory T and B cells) in the GBM microenvironment are also discussed [37].

Multiple myeloma (MM), an almost uncurable haematological malignancy, is characterized by the presence of plasma cells (PCs) in the bone marrow (BM). The presence of a high number of MM PCs in the peripheral blood has been found to correlate with disease progression and tumor relapse. In their review, Zeissig and coworkers address the current knowledge of the mechanisms underlying these processes, based on studies performed in in vitro, in vivo and clinical models. First, MM PCs are retained in the BM niche through their adhesion to bone marrow stromal cells (BMSCs), and this process is mediated by the interaction of MM PCs integrins/chemokine receptors with adhesion molecules produced by the BMSCs (i.e., the CXCL12 chemokine). Then, a significant decrease of these adhesion molecules, as well as an upregulation of matrix metalloproteinases, occurs and allows MM PCs to overcome these interactions and intravasate into the peripheral circulation to disseminate to new sites. MM PCs, travelling in the circulation, can reach distant sites where they extravasate and give rise to colonies in new BM niches; the chemokine CXCL12 is also deeply involved in this colonization process. This review highlights the current evidence supporting an association between chromosomal translocations frequent in MM patients (i.e., t(14;16), t(14;20) and t(4;14) translocations) and the incidence of metastasis formation. The authors also discuss the possible role of MM cell dissemination as a novel therapeutic target to overcome tumor relapse [38].

HMF (hypopigmented mycosis fungoides) is a form of cutaneous T-cell lymphoma (CTCL), characterized by specific features, such as light colored to achromic lesions, a dark skin phenotype, and predominance of immune CD8^+^ T-cells. The pathways of the pathogenesis for this type of lymphoma are discussed by Martinez Villarreal et al. In particular, these authors highlight that an active Th1/cytotoxic antitumor immune response, mediated for instance by the release of the TNF-α cytokine, is frequently detected in patients experiencing a favorable overall prognosis. Moreover, hypopigmentation represents a surrogate marker of cytotoxic immunity targeting cancer cells. The authors suggest that HMF might represent an intriguing model for the development of novel targeted therapeutic strategies to overcome tumor development and progression [39].

In conclusion, this Special Issue summarizes and discusses the most recent knowledge about the intricate biological networks underlying the processes of progression and, specifically, of cancer relapse in different tumors. The hopeful role of these discoveries in providing the basis for the development of novel and effective therapeutic approaches to overcome tumor drug resistance is also addressed. The authors of this Editorial also truly hope that this Issue might pave the way towards the translation from experimental studies into the clinical practice, in terms of precision medicine.
